# Changes in serum NO, ET-1, and VEGF after cannulated screw fixation in patients with femoral neck fractures and their relationship with femoral head necrosis

**DOI:** 10.3389/fphys.2025.1603323

**Published:** 2025-07-23

**Authors:** Ziqiang Li, Huanxi Wang, Tingwei Cao, Kewei Du

**Affiliations:** Department of Orthopedics, Shidong Hospital Affiliated to University of Shanghai for Science and Technology, Shanghai, China

**Keywords:** femoral head necrosis, femoral neck fractures, nitric oxide, endothelin-1, vascular endothelial growth factor

## Abstract

**Background:**

Femoral head necrosis (FHN) is one of the most serious complications in patients with femoral neck fractures (FNF) after cannulated screw fixation. Therefore, it is critical to predict the occurrence of FHN.

**Methods:**

FHN was diagnosed through clinical symptoms and imaging examinations. The serum levels of nitric oxide (NO), endothelin-1 (ET-1), and vascular endothelial growth factor (VEGF) in FNF patients were measured preoperatively and on postoperative days 3 and 5 using Enzyme-linked immunosorbent assay. The predictive value of NO, ET-1, and VEGF was evaluated using receiver operating characteristic curve analysis. The odds ratio (OR) for the risk factors of FHN was analyzed using multivariate logistic analysis.

**Result:**

The serum levels of NO and VEGF decreased post-surgery in patients with FNF, with a more pronounced decrease in those who subsequently developed FHN, whereas patients who did not develop FHN showed no significant changes in these levels. Conversely, the serum level of ET-1 increased after surgery in FNF patients, with a marked rise in those who experienced FHN, while no significant change was observed in patients without FHN. ROC analysis indicated that serum levels of NO, ET-1, and VEGF have predictive value for FHN occurrence in FNF patients, with the highest predictive accuracy observed on day 5 post-surgery (Serum NO had the AUC (95% CI) of 0.74 (0.67–0.81), 0.70 (0.62–0.78) for ET-1 and 0.73 (0.65–0.80) for VEGF, p < 0.001 for all). Operation time after fracture ≥48 h, Garden classification of III and IV, Panwels classification of III, Serum ET-1 at 3 days post operation >75.24 pg/mL were independent risk factors for FHN occurrence but Serum NO at 3 days post operation >35.98 nmol/mL, Serum NO at 5 days post operation >33.62 nmol/mL, Serum VEGF at 5 days post operation >66.45 pg/mL were protective factors.

**Conclusion:**

In patients with FNF who developed FHN, serum levels of NO and VEGF were reduced, while ET-1 levels were elevated, compared to those who did not develop FHN. Furthermore, on day 5 post-surgery, these three markers provided the strongest predictive value for the occurrence of FHN in FNF patients.

## Introduction

Femoral neck fractures (FNF) occur in the region between the femoral head and the base of the femoral neck, accounting for approximately 3.58% of all fractures. This type of fracture in young people represents only 3% of fractures in this region (Robinson et al., 1995). Treatment options for FNF include both conservative and surgical approaches. Conservative treatment is primarily suitable for non-displaced fractures, although this approach carries a higher risk (Taha et al., 2015). Currently, internal fixation remains the mainstay of treatment. Among these, cannulated screw fixation (CSF) is widely used due to its minimal invasiveness, reduced implant burden, and ease of operation (Lim et al., 2021). Although fixation can restore anatomical alignment and relieve vascular distortion, screw insertion may damage intraosseous blood vessels, reduce shear stress, and trigger endothelial apoptosis, ultimately leading to femoral head necrosis (FHN) (Xu et al., 2019). FHN is a major complication following internal fixation of femoral neck fractures in young and middle-aged adults, with reported incidence rates ranging from 10% to 45% (Davidovitch et al., 2010; Wu, 2010; Duckworth et al., 2011). A meta-analysis involving 41 studies showed that the incidence of FHN was 14.3% (Slobogean et al., 2015).

FHN can cause joint stiffness, limited mobility, and gait disturbance, often requiring secondary surgery and reducing quality of life. Currently, the precise pathogenesis of FHN remains unclear. However, vascular injury in the weight-bearing region of the femoral head may contribute to the development of FHN. Endothelin-1 (ET-1), a vasoconstrictor peptide, can influence vascular smooth muscle through paracrine and autocrine mechanisms (Brewster et al., 2020). By binding to ET receptors on cell membranes, ET-1 induces strong venous constriction, disrupting the blood supply to the femoral head. This disruption can lead to thrombosis, impaired blood flow, and ultimately FHN (Brewster et al., 2020). In contrast, nitric oxide (NO) supports angiogenesis and may protect against osteonecrosis (Calder et al., 2004).

Fracture healing strongly depends on angiogenesis, a critical step in the repair process. Adequate blood supply to the fracture site is vital for successful recovery. Multiple factors regulate neovascularization, including transforming growth factor, prostaglandin E, fibroblast growth factor, and Vascular endothelial growth factor (VEGF) (Hoffmann-Mlodzianowska et al., 2024). VEGF is essential for endothelial proliferation and angiogenesis, playing a key role in fracture healing by promoting blood vessel formation, regulating osteoclast activity, supporting cartilage maturation, and enhancing the interaction between chondrogenesis and osteoblasts (Zhang et al., 1997; Padubidri and Browne, 1996). It also contributes to bone formation and soft tissue repair. Previous studies have identified VEGF as a critical factor in the development of femoral head necrosis FHN (Xie and Huang, 2019). In light of these findings, this study focuses on VEGF, NO, and ET-1 in serum, analyzing the changes in their levels in patients with FNF before surgery and on days 3 and 5 post-operation. It also explores the relationship between these factors at different time points and the occurrence of FHN over a 2-year follow-up period post-surgery.

## Materials and methods

### Participants

This study was a retrospective analysis of 245 eligible patients with FNF who received hollow screw internal fixation. Over a 2-year follow-up period, 62 patients developed FHN, while 183 patients did not experience this condition, remaining classified as NFHN. The study was approved by Shidong Hospital Affiliated to University of Shanghai for Science and Technology. Informed written consents were obtained from the participants.

### Inclusion criteria

Patients were included in the study if they met all of the following criteria 1) Clinical diagnosis of a femoral neck fracture based on clinical symptoms and imaging findings; 2) Medical indication for open reduction and internal fixation and underwent hollow screw internal fixation as the primary surgical procedure; 3) unilateral femoral neck fracture; 4) age ≥18 years at the time of surgery; 5) Complete availability of clinical records and follow-up data; 6) informed consent obtained and signed.

### Exclusion criteria

Patients were excluded if they had any of the following conditions 1) The presence of infectious diseases, malignant tumors, hematological disorders, or autoimmune diseases; 2) Pre-existing hip osteoarthritis, synovitis, or other conditions that could lead to FHN; 3) Concomitant fractures at other sites; 4) Old FNF; 5) Long-term glucocorticoid therapy or chronic alcohol abuse; 6) Lost to follow-up within 2 years after hollow screw internal fixation.

### Diagnostic criteria for FHN

The diagnosis of FHN was made based on the criteria established in Surgery, 5th Edition. X-ray results revealed changes such as altered internal density of the femoral head, disorganized or sparse trabecular arrangement, presence of a crescent sign, femoral head collapse, joint space narrowing, discontinuity of the Shenton line, and osteoarthritic changes. Additionally, CT scans revealed early bone changes and collapse, while MRI identified the characteristic double-line sign, confirming FHN.

### Enzyme-linked immunosorbent assay (ELISA)

Serum samples were collected preoperatively and on postoperative days 3 and 5 for analysis. Serum levels of VEGF and NO were measured using ELISA kits. The VEGF kit was purchased from Abcam (ab222510, MA, United States), the ET-1 kit from Shanghai MLBio (ml025101, Shanghai, China), and the NO kit from Beyotime (S0024, Shanghai, China). All experimental procedures were conducted according to the manufacturer’s protocols.

### Statistics analysis

The data were presented using violin plots. Differences in significance were analyzed using the Brown-Forsythe ANOVA test followed with Games-Howell’s multiple comparisons test, unpaired t-test with Welch’s correction, Fisher’s exact test or Chi-square test (GraphPad software).

## Results

### Comparison of serum NO, ET-1, and VEGF levels preoperatively and at 3 and 5 days post-operation

**FIGURE 1:**
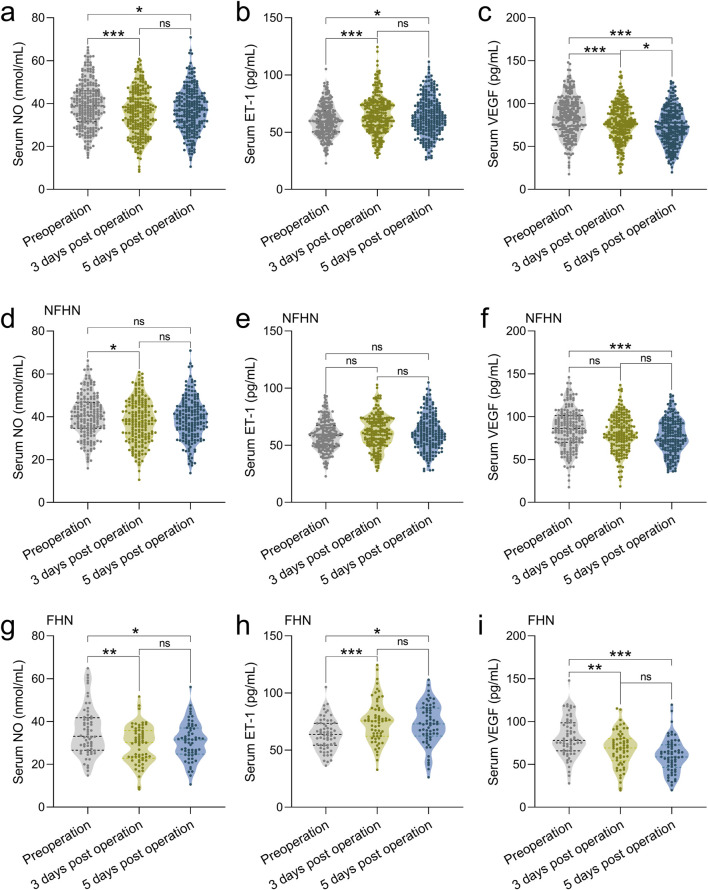
Comparisons of serum NO **(a)**, ET-1 **(b)** and VEGF **(c)** at the time of pre-operation, 3 days post-operation, 5 days post-operation in 245 patients with FNF who received the CSF. Comparisons of serum NO **(d)**, ET-1 **(e)** and VEGF **(f)** at the time of pre-operation, 3 days post-operation, 5 days post-operation in 183 patients with FNF received the CSF who did not undergo FHN during 2 years of follow-up. Comparisons of serum NO **(g)**, ET-1 **(h)** and VEGF **(i)** at the time of pre-operation, 3 days post-operation, 5 days post-operation in 62 patients with FNF received the CSF who underwent FHN (FHN) during 2 years of follow-up. Data were shown with violin plot. *p < 0.05, **p < 0.01, ***p < 0.001 and ns means no significance. Brown-Forsythe ANOVA test followed with Games-Howell’s multiple comparisons test.

Serum levels of VEGF, NO, and ET-1 were assessed in 245 patients with FNF undergoing cannulated screw fixation. Measurements were taken preoperatively and on postoperative days 3 and 5 to evaluate temporal changes in biomarker expression. The results showed significant decreases in serum NO and VEGF levels on day 3 post-operation (Figures 1a,c), along with a significant increase in ET-1 levels (Figure 1b). This trend persisted until day 5, with NO and VEGF levels remaining below their preoperative levels (Figures 1a,c). However, there were no significant differences in NO and ET-1 levels between days 3 and 5 post-surgery (Figures 1a,b). Notably, VEGF levels continued to decline on day 5 compared to day 3 (Figure 1c).

### Characteristics of clinical factors for FHN after CSF in patients with FNF during 2 years of follow-up

Among the 245 patients with FNF who underwent cannulated screw fixation, 62 developed FHN during the 2-year follow-up, while 183 did not (NFHN). Baseline comparisons between groups revealed no significant differences in age, sex, BMI, fracture side, injury mechanism, or comorbidities such as diabetes and hypertension (Table 1). However, FHN was significantly associated with delayed surgery (>48 h after fracture, *p* = 0.013), higher Garden classification (III–IV, *p* = 0.008), Pauwels type III fractures (*p* < 0.001), and a greater prevalence of smoking (*p* = 0.023) (Table 1). These factors may contribute to impaired vascular supply or delayed healing, thereby increasing the risk of FHN following internal fixation.

**TABLE 1:** Characteristics of clinical factors for femoral head necrosis (FHN) after cannulated screw fixation in patients with femoral neck fractures (FNF) during 2 years of follow-up.

Clinical factors	NFHN (n = 183)	FHN (n = 62)	p value
Age (years, %)			
<40	67 (36.6%)	20 (32.3%)	0.645
≥40	116 (63.4%)	42 (67.7%)	
Body mass index (kg/m^2^, %)			
<24	78 (42.6%)	24 (38.7%)	0.656
≥24	105 (57.4%)	38 (61.3%)	
Gender (n, %)			
Male	114 (62.3%)	40 (64.5%)	0.879
Female	69 (37.7%)	22 (35.5%)	
Fracture side (n, %)			
Left	109 (59.6%)	36 (58.1%)	0.882
Right	74 (40.4%)	26 (41.9%)	
Operation time after fracture (h, %)			
<48	96 (52.5%)	21 (33.9%)	0.013
≥48	87 (47.5%)	41 (66.1%)	
Cause of fracture (n, %)			
Low-energy trauma	99 (54.1%)	28 (45.2%)	0.242
High-energy trauma	84 (45.9%)	34 (54.8%)	
Location of fracture line (n, %)			
Subcapital	54 (29.5%)	23 (37.1%)	0.501
Transverse	103 (56.3%)	30 (48.4%)	
Basal	26 (14.2%)	9 (14.5%)	
Garden classification (n, %)			
I and II	105 (57.4%)	23 (37.1%)	0.008
III and IV	78 (42.6%)	39 (62.9%)	
Panwels classification (n, %)			
I	51 (27.9%)	10 (16.1%)	<0.001
II	89 (48.6%)	21 (33.9%)	
III	43 (23.5%)	31 (50.0%)	
Diabetes mellitus (n, %)			
Yes	37 (20.2%)	18 (29.0%)	0.162
No	146 (79.8%)	44 (71.0%)	
Hypertension (n, %)			
Yes	52 (28.4%)	21 (33.9%)	0.426
No	131 (71.6%)	41 (66.1%)	
Smoking (n, %)			
Yes	45 (24.6%)	25 (40.3%)	0.023
No	138 (75.4%)	37 (59.7%)	
Fixation remotion (n, %)			
Yes	64 (35.0%)	27 (43.5%)	0.229
No	119 (65.0%)	35 (56.5%)	

The data are presented as n (percentage). The comparisons of data between the two groups were done by Fisher’s exact test or Chi-square test.

### Comparisons of serum NO, ET-1, and VEGF at the time of pre-operation, 3 days post-operation, and 5 days post-operation among FHN and NFHN

We consequently analyzed serum VEGF, NO, and ET-1 levels in 62 patients who developed FHN and 183 who did not (NFHN), all of whom underwent cannulated screw fixation. Biomarker levels were measured preoperatively and on postoperative days 3 and 5 during a 2-year follow-up. In patients with NFHN, serum NO levels showed only a minimal decrease on postoperative days 3 and 5 compared to preoperative levels (Figure 1d), and ET-1 levels did not exhibit significant changes (Figure 1e). However, serum VEGF levels significantly decreased by postoperative day 5 relative to preoperative levels (Figure 1f). In contrast, in the 62 patients with FHN, serum NO levels remained lower than preoperative levels by postoperative day 5 (Figure 1g), and ET-1 levels continued to be elevated compared to preoperative levels (Figure 1h). Additionally, serum VEGF levels in FHN patients showed a rapid decline by postoperative day 3 (Figure 1i). Collectively, these findings suggest a close association between these three factors and the occurrence of FHN.

### Comparisons of serum NO between FHN and NFHN patients at the time of pre-operation, 3 days post-operation, 5 days post-operation

**FIGURE 2:**
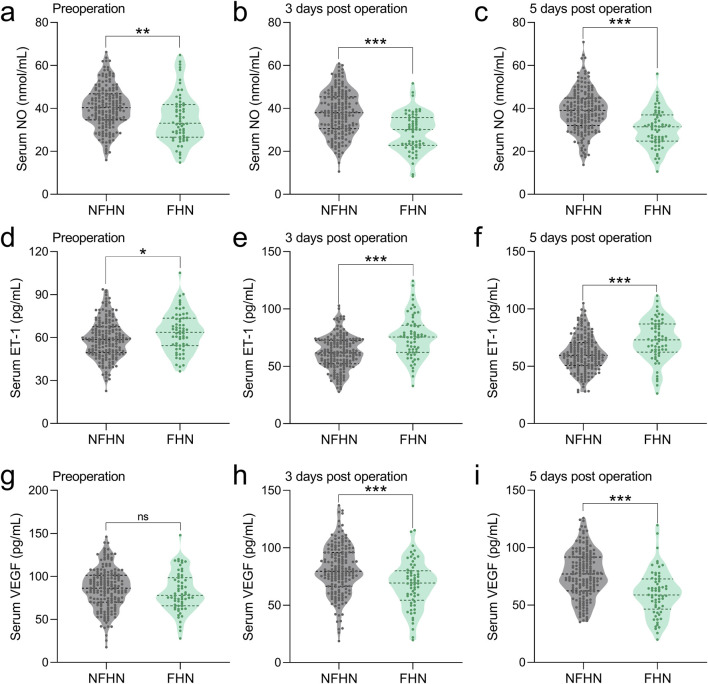
Comparisons of serum NO between FHN and NFHN patients at the time of pre-operation **(a)**, 3 days post-operation **(b)**, 5 days post-operation **(c)**. Comparisons of serum ET-1 between FHN and NFHN patients at the time of pre-operation **(d)**, 3 days post-operation **(e)**, 5 days post-operation **(f)**. Comparisons of serum VEGF between FHN and NFHN patients at the time of pre-operation **(g)**, 3 days post-operation **(h)**, 5 days post-operation **(i)**. Data were shown with violin plot. *p < 0.05, **p < 0.01, ***p < 0.001 and ns means no significance. Unpaired t-test with Welch’s correction.

We compared serum NO, ET-1, and VEGF levels at three time points between 62 patients who developed FHN and 183 who did not over a 2-year follow-up. Preoperatively, the FHN group showed significantly lower NO (Figure 2a) and higher ET-1 levels (Figure 2d), with no difference in VEGF (Figure 2g). On postoperative day 3, NO remained lower (Figure 2b), ET-1 higher (Figure 2e), and VEGF was significantly reduced in the FHN group (Figure 2h). These trends persisted on day 5, with consistently lower NO (Figure 2c), higher ET-1 (Figure 2f), and lower VEGF (Figure 2i) in FHN patients. Overall, FHN was associated with persistently decreased NO and VEGF and elevated ET-1 across all time points.

### ROC analysis of the predictive values of serum NO, ET-1, and VEGF

**FIGURE 3:**
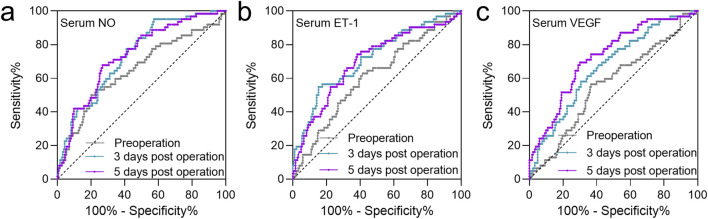
ROC analysis of the predictive values of serum NO **(a)**, ET-1 **(b)**, and VEGF **(c)** at the time of pre-operation, 3 days post-operation, 5 days post-operation for FHN after CSF in patients with FNF during 2 years of follow-up.

Previous studies have demonstrated a close association between NO, ET-1, and VEGF levels and the occurrence of FHN. In this study, the predictive value of preoperative and postoperative (3 and 5 days) serum levels of VEGF, NO, and ET-1 for FHN occurrence within 2 years was analyzed using ROC curves. The AUC values for preoperative serum NO, ET-1, and VEGF were 0.65 (0.57–0.737), 0.65 (0.57–0.737), and 0.56 (0.49–0.64), respectively (Figures 3a–c; Table 2). At 3 days post-operation, the AUC values for serum NO, ET-1, and VEGF increased to 0.74 (0.67–0.80), 0.74 (0.67–0.80), and 0.67 (0.59–0.74), respectively (Figures 3a–c; Table 2). Similarly, at 5 days post-operation, the AUC values for serum NO, ET-1, and VEGF were 0.74 (0.67–0.81), 0.74 (0.67–0.81), and 0.73 (0.65–0.80), respectively (Figures 3a–c; Table 2). These results indicate that the predictive value of serum NO, ET-1, and VEGF was higher at 3- and 5 days post-surgery than preoperatively.

**TABLE 2:** Predictive values in ROC analysis.

Serum Biomarkers	AUC (95% CI)	Cut off	Sensitivity%	Specificity%	p	Youden index
Preoperative serum NO	0.65 (0.57–0.737)	32.96 nmol/mL	50.00	79.78	<0.001	0.30
Preoperative serum ET-1	0.61 (0.53–0.69)	59.96 pg/mL	62.90	58.47	0.012	0.21
Preoperative serum VEGF	0.56 (0.49–0.64)	80.37 pg/mL	56.45	63.39	0.152	0.20
Serum NO at 3 days post operation	0.74 (0.67–0.80)	35.98 nmol/mL	77.42	59.02	<0.001	0.36
Serum ET-1 at 3 days post operation	0.72 (0.64–0.79)	75.24 pg/mL	54.84	84.7	<0.001	0.40
Serum VEGF at 3 days post operation	0.67 (0.59–0.74)	72.15 pg/mL	58.06	69.40	<0.001	0.27
Serum NO at 5 days post operation	0.74 (0.67–0.81)	33.62 nmol/mL	67.74	73.22	<0.001	0.41
Serum ET-1 at 5 days post operation	0.70 (0.62–0.78)	63.64 pg/mL	74.19	61.75	<0.001	0.36
Serum VEGF at 5 days post operation	0.73 (0.65–0.80)	66.45 pg/mL	69.35	69.95	<0.001	0.40

CI: confidence interval.

### Multivariate logistic analysis of predictors for FHN after CSF in patients with FNF during 2 years of follow-up

A multivariate analysis was conducted to identify independent risk factors for FHN in patients with FNF over a 2-year follow-up. FHN occurrence was set as the dependent variable (1 = FHN, 0 = NFHN), with significant clinical variables from Table 1 and serum levels of VEGF, NO, and ET-1 at different time points included as independent variables. Delayed surgery (>48 h post-fracture) was associated with a higher risk of FHN (OR = 1.65, *p* = 0.008, Table 3), as were higher Garden (III–IV) (OR = 2.17, *p* < 0.001) and Pauwels (type III) classifications (OR = 1.75, *p* = 0.006). In terms of serum biomarkers, elevated serum ET-1 levels (>75.24 pg/mL) on postoperative day 3 were associated with an increased risk of FHN (OR = 1.32, p = 0.021, Table 3). Conversely, higher serum NO levels on postoperative day 3 (>35.98 nmol/mL) and day 5 (>33.62 nmol/mL) were associated with a reduced risk of FHN (OR = 0.88, p = 0.036, and OR = 0.85, p = 0.012, respectively) (Table 3). Furthermore, elevated serum VEGF levels on day 5 (>66.45 pg/mL) were also found to be protective against FHN (OR = 0.87, p = 0.009, Table 3). In summary, delayed surgery, higher Garden and Pauwels classifications, and elevated serum ET-1 levels on postoperative day 3 were identified as independent risk factors for FHN. In contrast, higher serum NO and VEGF levels on days 3 and 5 post-surgery appeared to act as protective factors. These findings highlight the potential predictive value of these clinical and biochemical markers for assessing the risk of FHN in femoral neck fracture patients.

**TABLE 3:** Multivariate logistic analysis of predictors for femoral head necrosis (FHN) after cannulated screw fixation in patients with femoral neck fractures (FNF) during 2 years of follow-up.

Predictors	OR	95% CI	p value
Operation time after fracture ≥48 h	1.65	1.16 to 2.29	0.008
Garden classification: III and IV	2.17	1.56 to 3.34	<0.001
Panwels classification: III	1.75	1.29 to 2.81	0.006
Serum NO at 3 days post operation >35.98 nmol/mL	0.88	0.82 to 0.95	0.036
Serum ET-1 at 3 days post operation >75.24 pg/mL	1.32	1.07 to 1.93	0.021
Serum NO at 5 days post operation >33.62 nmol/mL	0.85	0.79 to 0.96	0.012
Serum VEGF at 5 days post operation >66.45 pg/mL	0.87	0.77 to 0.94	0.009

OR: odds ratio, CI: confidence interval.

## Discussion

FHN is a serious complication following internal fixation of FNF, with a growing incidence. Despite its clinical importance, the pathophysiological mechanisms underlying FHN remain incompletely understood. In this study, we identify a strong association between serum levels of NO, ET-1, and VEGF and the subsequent development of FHN. These biomarkers may offer predictive value for risk stratification, enabling clinicians to identify high-risk patients at the time of surgical planning. Several studies have investigated risk factors for FHN after FNF, though findings remain inconsistent. Razik et al. retrospectively analyzed 92 intracapsular femoral neck fracture cases with a mean follow-up of 2 years, stratified by time to surgery (Upadhyay et al., 2004). They found no significant difference in FHN incidence between patients operated on within 48 h and those treated later (Upadhyay et al., 2004). In 2015, a systematic review of seven articles found insufficient evidence to establish a relationship between the timing of surgery and FHN (Papakostidis et al., 2015). In contrast, our results show a significantly higher FHN incidence in patients operated on more than 48 h after injury, aligning with studies suggesting that delayed fixation may elevate intracapsular pressure and impair femoral head perfusion (Wu and Cao, 2002). These discrepancies may reflect differences in patient selection, fracture characteristics, or surgical protocols.

Fracture displacement and biomechanics are well-established contributors to vascular injury. Consistent with previous findings (Li et al., 2024; Olansen et al., 2024), we observed higher FHN risk in patients with greater Pauwels and Garden classifications, underscoring the importance of timely and accurate anatomical reduction to preserve blood supply.

ET-1 is a potent vasoconstrictor that increases calcium influx and potentiates the effects of other vasoconstrictors such as catecholamines and angiotensin. This leads to reduced local perfusion, resulting in ischemia, endothelial damage, intravascular coagulation, and compromised blood supply to the femoral head (Zhang and Peng, 2017). Our findings indicate that elevated serum ET-1 levels are associated with a higher risk of postoperative FHN in patients with femoral neck fractures. ET-1 levels were significantly higher in patients who developed FHN and continued to rise through day 5 after surgery, whereas levels remained stable in those without FHN. NO plays a crucial role in regulating osteocyte apoptosis by combining with superoxide anions to lower oxygen-free radical levels, thereby preventing damage to microvessels and synovium (Bao et al., 2012). Another study found significantly higher levels of endothelial NO synthase in osteoblasts, osteoclasts, and bone marrow cells of patients with bone necrosis than in those with osteoarthritis (Calder et al., 2004). In our study, NO levels were significantly lower in patients with FHN than in non-FHN patients, supporting a negative correlation between NO and bone necrosis. Further analysis revealed that NO levels in FNF patients who developed FHN were lower postoperatively than preoperative levels, indicating that reduced NO levels may be closely linked to the development of FHN.

VEGF, a specialized growth factor that drives endothelial cell proliferation and migration, plays a vital role in promoting vascular invasion during chondrogenesis and bone healing (Ferrara and Davis-Smyth, 1997; Poltorak et al., 1997). Wu et al. reported that gene expression in bone callus varies throughout fracture healing (Wu and Wang, 2001). In the early phase (days 5–7), reduced VEGF expression was observed, possibly due to disrupted local blood flow from bleeding, cell necrosis, and inflammation (Wu and Wang, 2001). Our analysis also revealed similar findings, showing that VEGF levels decreased by day 5 post-surgery in patients with FNF who developed FHN. Wu et al. further found that during fracture healing, rapid granulation tissue growth can reduce local blood flow and oxygen tension, strongly inducing VEGF expression through hypoxia. Therefore, it is important to monitor the level of VEGF in patients with FNF who do not develop FHN for an extended period after surgery.

Dou et al. showed that serum NO and ET-1 levels on postoperative day 1, rather than days 3 or 5, had stronger predictive value for FHN, suggesting early biomarker monitoring may help identify high-risk patients (Dou et al., 2021). In contrast, our analysis revealed that NO and ET-1 levels measured 5 days after surgery provided the strongest predictive value for FHN occurrence. Although previous studies have not evaluated VEGF as a predictor of FHN, our findings show that postoperative VEGF levels on days 3 and 5—not preoperative levels—effectively predicted FHN in patients with femoral neck fractures. These findings suggests that ET-1, NO, and VEGF may serve as a valuable biomarker in the postoperative phase, highlighting a close relationship between these three factors and FHN occurrence in FNF patients.

While early postoperative biomarker assessments—such as on day 1—are often emphasized in previous studies, our findings suggest that levels measured on days 3–5 may more accurately reflect ongoing vascular injury risk (Gwenzi et al., 2023). This difference may relate to cohort-specific factors, including fracture type [e.g., higher soft tissue damage in calcaneal avulsion fractures (Wan et al., 2024)], fixation method [intra-vs. extra-articular approaches (Zhao, 2023)], or systemic inflammation [e.g., the prognostic value of the systemic immune-inflammation index on day 5 in hip fracture patients (Celen, 2024)]. These results highlight the need to individualize biomarker monitoring based on biological events, such as the inflammatory peak around day 14 (Working et al., 2021), and the clinical context, including the timing of vascular repair relative to fixation (Tosun, 2020; Kriechling et al., 2024). Single timepoint measurements may therefore be insufficient for accurate risk assessment.

The interplay between NO, ET-1, and VEGF may represent a coordinated pathological network driving vascular and metabolic dysfunction in FHN. Elevated ET-1, a potent vasoconstrictor, suggests sustained hypoxia and impaired vascular repair. Conversely, reduced NO, an endothelial protector, indicates compromised vascular function and oxidative stress. Early postoperative suppression of VEGF, a key angiogenic and osteogenic factor, points to inadequate neovascularization during healing. Together, increased ET-1 and reduced NO and VEGF may serve as a pathological signature of failed revascularization in FHN. Notably, the predictive value of these biomarkers peaked on postoperative day 5, probably reflecting active vascular remodeling. These findings support the application of serial biomarker monitoring for improved risk stratification and underscore the need for prospective, time-resolved studies to define optimal surveillance windows.

Our study aimed to identify risk factors predictive of AVN following femoral neck fracture fixation, rather than to advocate early diagnosis or immediate surgical intervention. By establishing a predictive model, our findings support early risk stratification, particularly during surgical planning, to guide fixation choices and perioperative management in high-risk patients. Although AVN typically does not affect fracture healing and is managed after union, early identification of high-risk patients may allow for more tailored strategies, including alternative fixation methods, enhanced vascular protection, or closer imaging surveillance. These results should inform risk-based clinical decision-making, rather than suggest changes to surgical timing. Integrating prediction models into routine practice may ultimately improve long-term outcomes in patients undergoing internal fixation for femoral neck fractures.

This study has several limitations. First, its retrospective design introduces potential biases, including selection bias and incomplete data. Key surgical variables, such as reduction quality, fixation method, timing, and intraoperative details, were unavailable, limiting the accuracy of the predictive model. Moreover, the single-center setting may affect generalizability, and biomarker measurements at only three time points may have missed important dynamic changes. Additionally, the absence of an external validation cohort further limits the model’s applicability. Prospective, multicenter studies with standardized surgical data and broader clinical profiling are needed to confirm and strengthen these findings.

## Conclusion

Our study demonstrated that, over time after surgery, NO and VEGF levels decreased, whereas ET-1 significantly increased in FNF patients. Importantly, these three factors had an effective prediction effect on the FHN occurrence. However, our study still has some limitations. Given that ET-1, NO, and VEGF levels on day 5 post-surgery showed the strongest predictive value for FHN, further investigation into the combined predictive power of these factors is warranted. Additionally, these factors should be monitored over an extended period until fracture healing is complete to assess any reversal in trends.

## Data Availability

The original contributions presented in the study are included in the article/supplementary material, further inquiries can be directed to the corresponding author.
